# Step-by-Step Guide to Hematoma Prevention After Rhytidectomy: Lessons Learned from 40 Years of Experience

**DOI:** 10.1007/s00266-025-05235-7

**Published:** 2025-09-19

**Authors:** Niccolò Lazzeri Domar, Giovanni Botti, Chiara Botti

**Affiliations:** 1 Department of Plastic and Reconstructive Surgery, Catholic University of Sacro Cuore, University Hospital A. Gemelli, Rome, Italy; 2 Villa Bella Clinic, Salò, Italy

**Keywords:** Rhytidectomy, Hematoma prevention, Tranexamic acid, Fibrin glue, Hemostatic net, High SMAS/Extended SMAS facelift

## Abstract

**Background:**

Postoperative hematoma remains one of the most significant complications in rhytidectomy, with reported rates up to 15%. This study presents a structured, experience-based protocol for hematoma prevention, developed over 40 years and applied to over 3000 cervicofacial lift procedures. The protocol combines strict patient selection, advanced surgical technique, and perioperative management strategies aimed at minimizing bleeding risks.

**Methods:**

A retrospective analysis was conducted on a patient population of approximately 3000 individuals, with an emphasis on the most recent 500 cases performed under a unified protocol. Key preoperative measures include blood pressure control and avoidance of medications and supplements that increase bleeding risk. Intraoperative strategies involve infiltration with tranexamic acid (TXA), use of fibrin sealants (ARTISS), hemostatic net placement, and meticulous SMAS flap dissection and fixation using a modified High SMAS/Extended SMAS technique. Postoperative care focuses on pain, anxiety, and blood pressure control through long-acting anesthetics and appropriate dressings.

**Results:**

The systematic adoption of this multimodal approach has reduced the hematoma rate to approximately 0.3% over the past five years. The combination of TXA, fibrin glue, hemostatic nets, and blood pressure management has proven synergistically effective, despite the lack of randomized controlled comparisons.

**Conclusions:**

While the study is limited by its retrospective design and absence of a formal control group, the large sample size and low complication rate offer compelling evidence. This protocol represents a reproducible and practical guide for surgeons aiming to minimize hematoma risk in facelift surgery, supporting safer outcomes and faster recovery.

- A standardized, multimodal protocol has reduced hematoma rates in facelift surgery to 0.3%.
- The protocol integrates TXA infiltration, fibrin glue, hemostatic nets, and precise blood pressure control.
- Over 3000 facelift cases and 40 years of experience support the safety and reproducibility of the approach.
- This guide provides practical steps that can be directly applied to improve surgical outcomes in rhytidectomy.

**Level of Evidence III:**

This journal requires that authors assign a level of evidence to each article. For a full description of these Evidence-Based Medicine ratings, please refer to the Table of Contents or the online Instructions to Authors www.springer.com/00266.

## Introduction

Facelift surgery has been performed since the mid-1910s. [1] In over a century, its primary objectives have remained consistent: to achieve long-lasting facial rejuvenation while preserving the patient's natural identity.

Following Skoog’s deep plane technique [2], the authors adopt a modified High SMAS/Extended SMAS approach—described by Barton and refined by Marten [3, 4]—which enables the elevation of two distinct yet interconnected flaps in the medial cheek. These flaps can be repositioned along different vectors, allowing for individualized and natural results by respecting individual facial features.

**Fig. 1:**
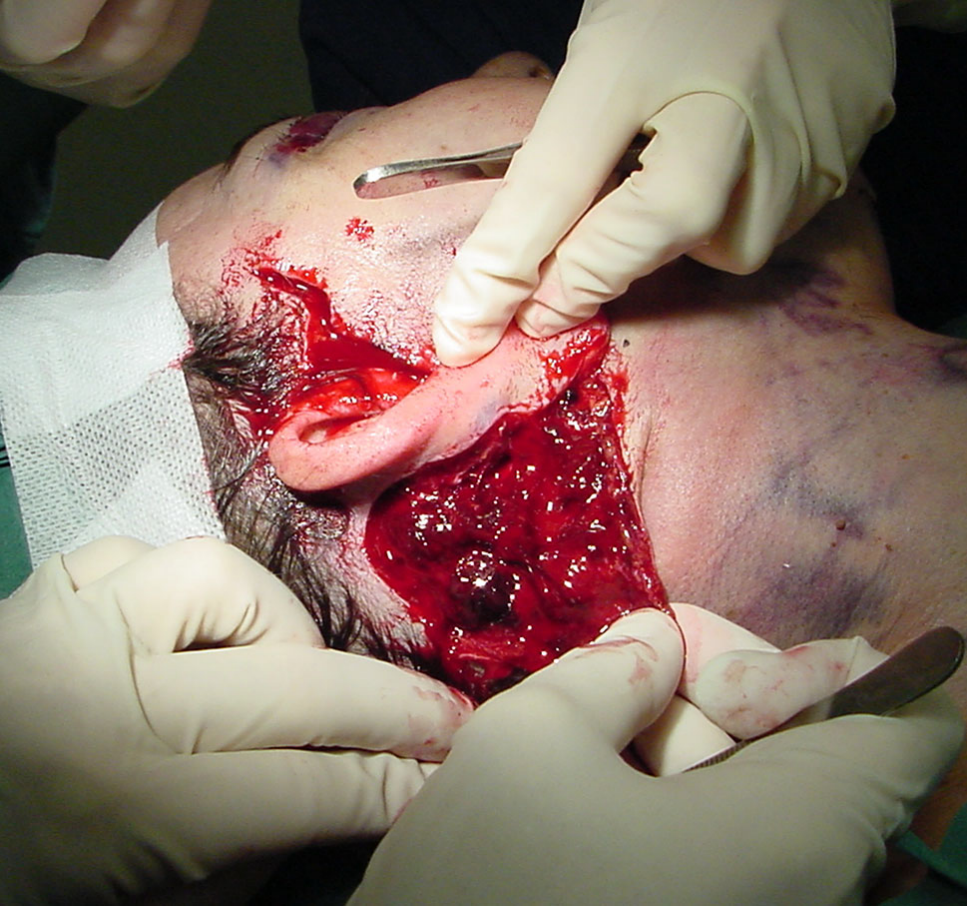
Postoperative hematoma localized in the postero-inferior auricular region following a facelift procedure. This area represents the most frequent site for hematoma formation due to its anatomical predisposition to blood accumulation

Among the most feared and unpredictable complications is postoperative hematoma (Fig. 1), which may significantly compromise both aesthetic results and patient safety. Reported incidence in the literature ranges from 0.2 to 15%. [5, 6]

This article outlines the authors’ preoperative, intraoperative, and postoperative strategies to minimize the occurrence of hematoma. Through strict adherence to these protocols, the authors have achieved a marked reduction in hematoma rates, currently around 0.3%. This guide offers practical insights to help surgeons achieve safer, more predictable outcomes in facelift surgery.

## Materials and Methods

### Patient Population and Data Analysis

Over the past 40 years, the two primary surgeons (G.B. and C.B.) have performed approximately 3000 cervicofacial lifting procedures. In the initial two decades, the average number of surgeries was approximately 50 per year, increasing to over 100 annually between 2005 and 2024. The cohort consisted of 85% female patients (≈2500) and 15% male patients (≈500), with a mean age of 57 years (range: 44–82). Most patients exhibited a normal body habitus; a retrospective analysis estimated a BMI of 22.5 kg/m^2^ (range: 19.5–27), with few obese or underweight individuals.

Patient selection adhered to strict inclusion criteria. Active smokers were excluded from surgical candidacy. Only patients with well-controlled blood pressure were considered eligible; in cases of hypertension, pharmacological stabilization was mandatory prior to surgery. Individuals with known coagulopathies were excluded. Patients receiving anticoagulant therapy were considered eligible only if temporary discontinuation of the medication was possible. In such cases, a tailored washout period was ensured to minimize the risk of intraoperative and postoperative bleeding.

### Surgical Technique

The authors employ a modified High SMAS/Extended SMAS facelift technique, described by Barton and refined by Marten. [3, 4] All procedures are performed under general anesthesia. Each hemiface is infiltrated with 100–120 cc of local anesthetic solution, consisting of 220 cc of saline solution, 20 cc of 3% mepivacaine, 1 mg of adrenaline, and 500 mg of tranexamic acid (TXA).

The anterior skin incision begins at the level of the temporal hairline and descends toward the root of the helix. In female patients, the incision follows the edge of the tragus, while in male patients, a pre-tragal course is preferred to avoid postoperative beard hair growth within the conchal region. The incision continues around the earlobe and ascends along the retro-auricular groove, maintaining an approximate distance of 5 mm from the conchal side to minimize scar visibility. It then arches posteriorly at the level of the anterior crus of the antihelix and extends along the occipital hairline for approximately 8–10 cm.

**Fig. 2:**
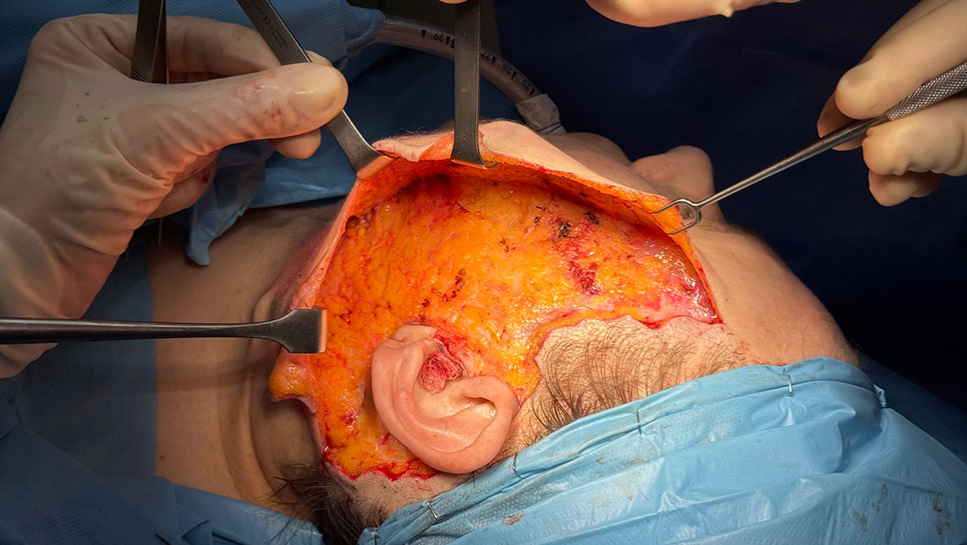
Intraoperative demonstration of the subcutaneous dissection extent, which is adjusted based on facial soft tissue laxity. In the cervical region, dissection typically extends 6–7 cm laterally from the midline

Subcutaneous dissection is performed using either cold instruments (blade and scissors) or electrocautery. The extent of dissection varies according to the degree of soft tissue laxity. In the cervical region, dissection typically extends 6–7 cm laterally from the midline (Fig. 2).

**Fig. 3:**
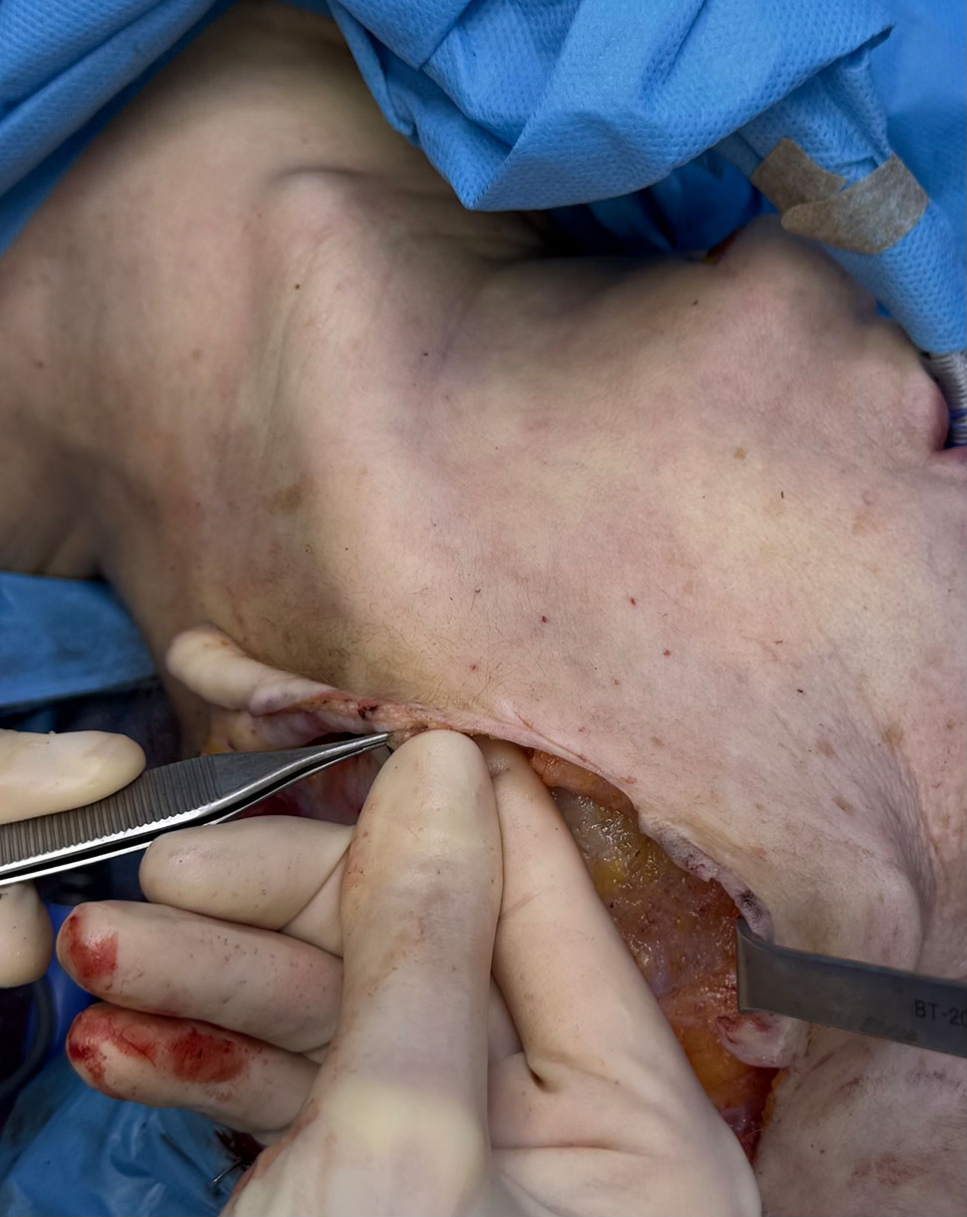
Intraoperative view of the sub-SMAS dissection at the cervical level. This maneuver facilitates neck contouring and enhances the definition of the cervico-mandibular angle

Prior to SMAS flap reshaping, a thin strip of SMAS is excised anterior to the ear to preserve the natural preauricular concavity. The superior incision of the SMAS flap begins on the lateral aspect of the malar bone and extends posteriorly above the zygomatic arch, where the temporo-frontal branch of the facial nerve lies in a deep, protected plane just above the periosteum. The vertical incision of the SMAS flap is placed approximately 2 cm anterior to the tragus and directed caudally toward the cervical region. Sub-SMAS dissection begins at the lateral end of the superior incision, reaching the anterior border of the masseter muscle. When needed, dissection can be extended medially to the nasolabial fold. In the cervical portion, sub-SMAS dissection is performed deep to the platysma. Tunnels are created using Trepsat scissors, then connected with scissors or electrocautery. Dissection extends to the midline to ensure sufficient flap mobility (Fig. 3). Special care is taken to preserve all facial nerve branches. [7]

**Fig. 4:**
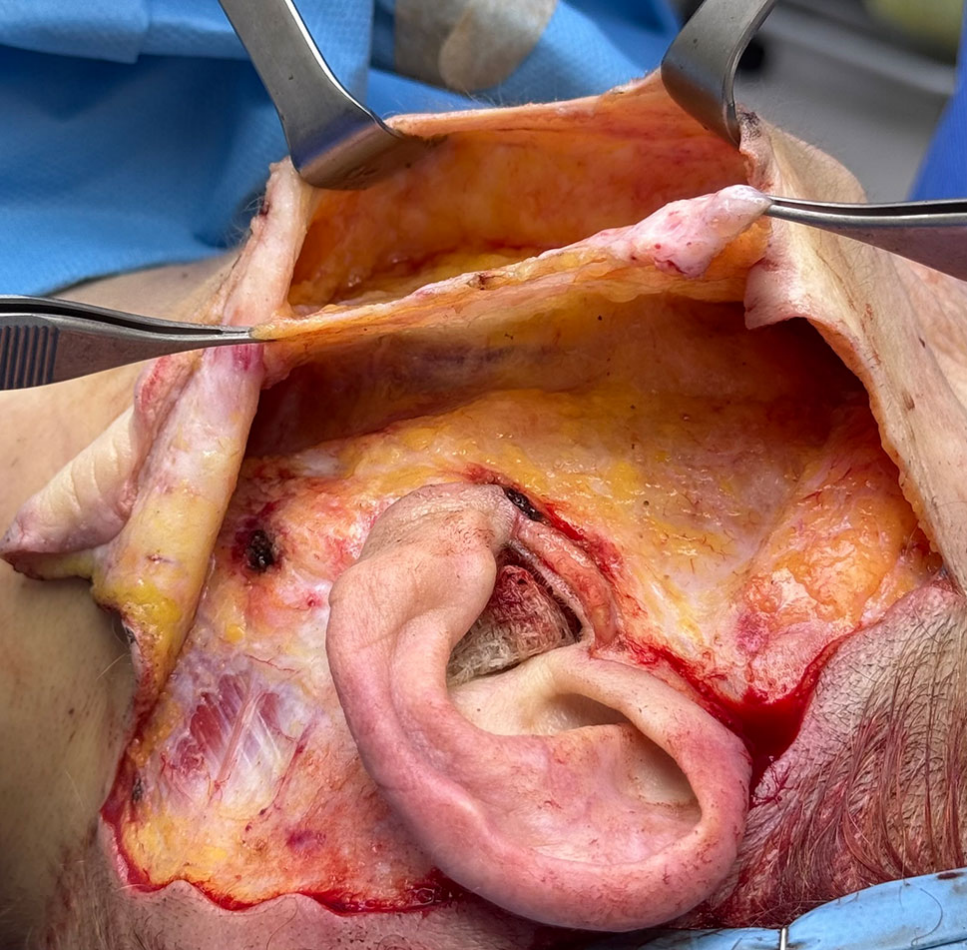
Intraoperative view of the fully mobilized and isolated SMAS flap

Once the SMAS flap is fully mobilized (Fig. 4), it is tractioned using two separate vectors: in the cheek the superior fascial component is pulled vertically, while in the neck, the muscular component is directed supero-laterally toward the retro-auricular area. To facilitate vertical elevation of the fascial component, a “back-cut” is made at the level of the earlobe.

For durable outcomes, anchoring the SMAS flap to stable and immobile structures is essential. These include the mastoid periosteum, Lore’s fascia, deep temporal fascia, and periosteum of the periorbital region and zygomatic arch.

After securing the SMAS flap, the adipo-cutaneous flap of the cheek is redraped along a transverse vector, carefully adjusted to prevent "dog-ears" formation at the upper end of the incision. The lower (cervical) adipo-cutaneous flap is pulled supero-laterally and then rotated supero-medially to effectively close the retro-auricular space.

**Fig. 5:**
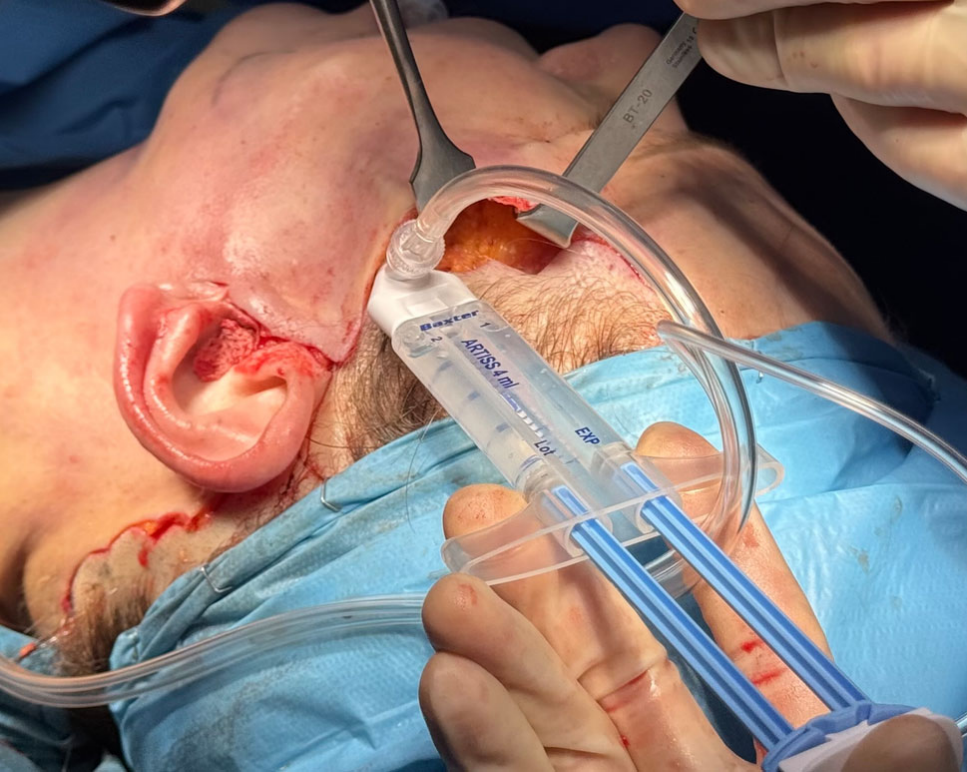
Application of ARTISS fibrin glue (Baxter Healthcare Corp., Deerfield, Illinois; 4 mL) between the adipo-cutaneous flap and the SMAS flap, immediately prior to skin closure

Once the skin has been secured with key sutures, fibrin glue (ARTISS, Baxter Healthcare Corp, Deerfield, Illinois) is applied between the adipo-cutaneous and underlying SMAS flaps to promote adhesion and minimize shear forces (Fig. 5).

An optional step, depending on the patient’s needs, involves the placement of a transcutaneous mesh (Auersvald type) in the retro-auricular region, where hematomas are most likely to form.

**Fig. 6:**
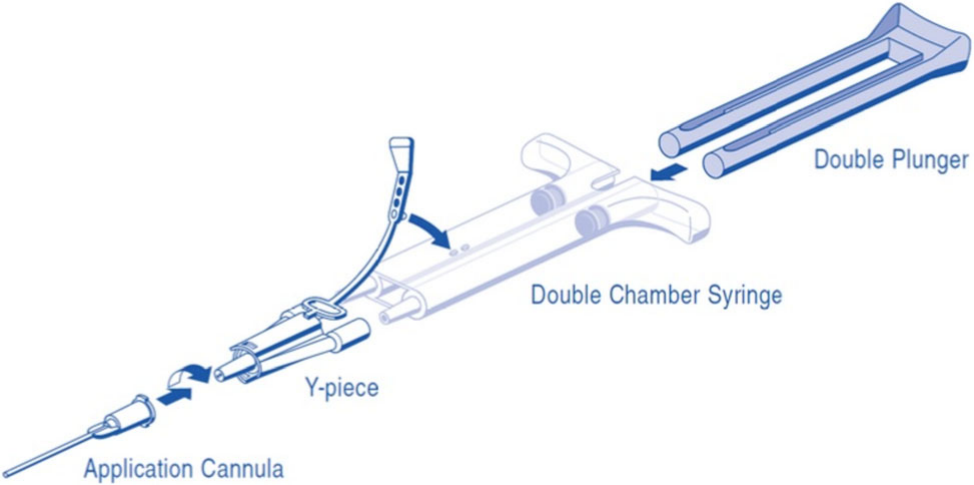
ARTISS fibrin glue (Baxter Healthcare Corp., Deerfield, Illinois; 4 mL)

Finally, a mildly compressive dressing is applied and removed within 24 h. (Fig. 6).

## Discussion

Postoperative hematoma is widely recognized as the most feared complication of cervicofacial lifting procedures, with reported incidence rates in the literature of 0.2–15%. [5, 6] Hematoma formation can compromise both aesthetic outcomes and patient safety.

All rhytidectomy techniques inherently alter the vascular supply to the adipo-cutaneous flap; thus, the accumulation of blood—particularly under pressure—may impair venous and lymphatic drainage, leading to tissue ischemia, prolonged swelling, and a self-perpetuating cycle of worsening edema. Additional complications may include infection, skin hyperpigmentation, delayed wound healing, neuropraxia, pathological scarring, respiratory distress, and extended recovery times. [8]

Numerous risk factors have been associated with hematoma development: male gender, elevated BMI (often linked to comorbidities such as hypertension and diabetes, as well as increased intrinsic surgical risk [9]), smoking, diabetes [10], combined surgical procedures performed in a single session [10], hypertension (particularly postoperative hypertensive peaks [11, 12]), hypotensive anesthesia, platysmaplasty, anticoagulant and/or antiplatelet therapy, and certain herbal supplements (e.g., ginkgo, garlic, and ginseng, known as the "three Gs"). [8] Advanced age has also been proposed as a risk factor, although findings in the literature remain inconclusive. [13–15]

Male patients are at increased risk, likely due to greater facial skin perfusion associated with denser capillary networks, possibly linked to the presence of facial hair. [16] Grover et al. reported that platysma plication increases hematoma risk by 4.3 times. [17]

Some could argue that dual-plane facelift techniques, involving both subcutaneous and sub-SMAS dissection, may increase bleeding risk. However, our clinical experience suggests otherwise. The sub-SMAS plane is a virtually avascular, gliding anatomical plane where dissection is safe and straightforward. Caution is advised only during the release of retaining ligaments (e.g., malar or masseteric cutaneous ligaments), where occasional vessels may require coagulation. Alternatively, as practiced by one of the authors (C.B.), dissection using electrocautery can further reduce the need for secondary hemostasis. In our extensive series, only one hematoma was documented within the sub-SMAS plane, confirming its excellent safety profile.

To date, no single strategy has demonstrated universal efficacy in preventing postoperative hematoma following facelift surgery. [18] Rather than assessing isolated measures, this study proposes a comprehensive, synergistic protocol combining multiple intraoperative and perioperative strategies. Although this is not a formal comparative study, the introduction of our current protocol has been associated with a marked reduction in hematoma rates: internal retrospective data show a historical incidence of 3–4% prior to its adoption, which has progressively declined over the past decade to approximately 0.3%, representing a clinically relevant improvement.

### Preoperative Phase

In the two weeks preceding surgery, patients must avoid medications that increase bleeding risk, including aspirin, nonsteroidal anti-inflammatory drugs (NSAIDs), and specific herbal supplements. For individuals on anticoagulant or antiplatelet therapy, temporary discontinuation must be evaluated in coordination with the general practitioner.

Optimal blood pressure control is critical. To minimize perioperative hypertensive spikes, all patients receive a 5 mg transdermal clonidine patch 24 h before surgery. Clonidine, an alpha-2 adrenergic agonist, acts centrally by inhibiting norepinephrine release, thereby reducing blood pressure and providing anxiolytic and analgesic effects. [19] Its long-acting profile is particularly useful in preventing episodes of "reactive hypertension," as described by Berner et al. [20], which typically occur 3–5 h after rhytidectomy and represent a significant risk factor for postoperative hematoma.

### Intraoperative Phase

All patients undergo infiltration with approximately 100–120 cc per side of a solution composed of 220 cc saline, 20 cc of 3% mepivacaine, 1 mg epinephrine, and 500 mg of TXA.

TXA complements epinephrine in reducing intraoperative bleeding through a different mechanism of action. As an antifibrinolytic agent, TXA inhibits plasminogen activation, thereby stabilizing clot formation and reducing blood loss. [21] Multiple studies have confirmed its efficacy in decreasing complications associated with rhytidectomy. [22, 23] A systematic review by Brown and Rohrich consolidated evidence supporting TXA’s role in reducing intraoperative blood loss, surgical time, drainage volume [24], and postoperative erythema by inhibiting neovascularization. [25]

The combination of dilute local anesthesia, epinephrine, and TXA induces vasoconstriction, which delays and prolongs TXA absorption, theoretically prolonging its antifibrinolytic effect. [26]

Although concerns have been raised regarding the potential flap ischemia associated with TXA, current evidence suggests that this risk is more likely linked to overly tight transdermal meshes rather than the drug itself.

A pivotal step in hematoma prevention is the application of fibrin glue (fibrin sealant), which serves to eliminate dead space while providing direct hemostatic action. [18]

First introduced during World War I by Grey for topical hemostasis [27], fibrin glue has since been widely adopted across multiple surgical specialties. [28–30] In aesthetic surgery, its adoption remains limited, likely due to cost considerations.

Fibrin glue replicates the final stage of the coagulation cascade, with separate components—fibrinogen and factor XIII in one chamber, and thrombin in the other—preserved until mixed.

Fibrin sealants improve tissue adhesion to the wound bed, thereby minimizing shearing, reducing ecchymosis, and obliterating potential dead space beneath the skin where fluid may otherwise accumulate. These properties significantly reduce the risk of postoperative hematomas, as reported in multiple studies. [18, 31–37] Moreover, the use of fibrin glue allows for the elimination of surgical drains, which have never been shown to significantly reduce hematoma rates. [11, 31] Conversely, drains are associated with patient discomfort and several potential complications, including infection, vascular injury during removal, peripheral nerve damage, local pressure on the flap (which can lead to necrosis), painful removal, and prolonged nursing care. [37]

In 1989, Botti et al. reported a hematoma rate of just 1% in a series of 40 facelift procedures using fibrin sealants, compared to 25% in previous cases without their use. [38] The product currently employed, ARTISS (Baxter Healthcare Corp.), has been approved for rhytidectomy since 2011. [39] ARTISS is a dual-component, plasma-derived sealant containing aprotinin, factor XIII, fibrinogen, thrombin, and calcium chloride. Before application, the surgical field must be adequately dried. The applicator system (Spray Set; Baxter) and pressure regulator (Easyspray; Baxter) enable a fine mist delivery, creating a thin layer of product. Its low thrombin concentration delays polymerization, offering up to 60 s for precise flap repositioning after application.

Once the adipo-cutaneous flap is correctly positioned, gentle medial-to-lateral compression should be applied to remove any excess product. The flap should then be held in place with light compression for at least 3 min to ensure proper solidification of ARTISS and stable adhesion to the underlying tissues. [40]

The senior authors (G.B. and C.B.) have employed fibrin sealants consistently throughout their careers, with the exception of a temporary suspension in the early 2000s due to concerns over bovine spongiform encephalopathy (BSE), which led to the temporary withdrawal of certain fibrin-based products.

To further reduce hematoma risk, a transcutaneous hemostatic net (Auersvald type) is placed in the postero-inferior auricular region, where blood collections are most common (as the use of fibrin glue limits its necessity). [41] This net prevents hematoma formation within the first 72 postoperative hours by obliterating dead space, exerting pressure between the skin and SMAS–platysma layers, and enhancing flap stability. [41] Literature supports its efficacy without increasing the risk of ischemia or necrosis. [41, 42]

In our experience, no hematomas were observed following removal of the hemostatic net.

Intraoperative blood pressure management is also crucial. The association between hypertension and hematoma formation was first described by Berner et al. in 1976 [20] and has since been corroborated by multiple studies. Restoring normotension before achieving final hemostasis (which still is a crucial step for hematoma prevention) and wound closure is essential to avoid bleeding complications. [43, 44]

The intraoperative use of drugs such as propofol, dexamethasone, ondansetron, promethazine, and metoclopramide has been shown to reduce postoperative nausea and vomiting, indirectly lowering the risk of hematoma formation. [8] Additionally, a smooth awakening from general anesthesia is essential, as agitation, retching, Valsalva maneuvers, and hypertensive spikes during awakening can contribute to bleeding. Although controversial and potentially risky, extubating the patient just before full awakening may help mitigate these reactions and reduce related complications.

Despite the use of fibrin sealant and/or hemostatic nets, proper postoperative dressing is still important. A moderately compressive bandage is applied to cover all areas of dissection of the adipo-cutaneous flap across the face and neck and is removed on the first postoperative day.

### Postoperative Phase

Postoperative control of pain, nausea, and anxiety is essential, particularly within the first 24 h, to prevent hypertensive peaks that may predispose to hematoma formation.

**Fig. 7:**
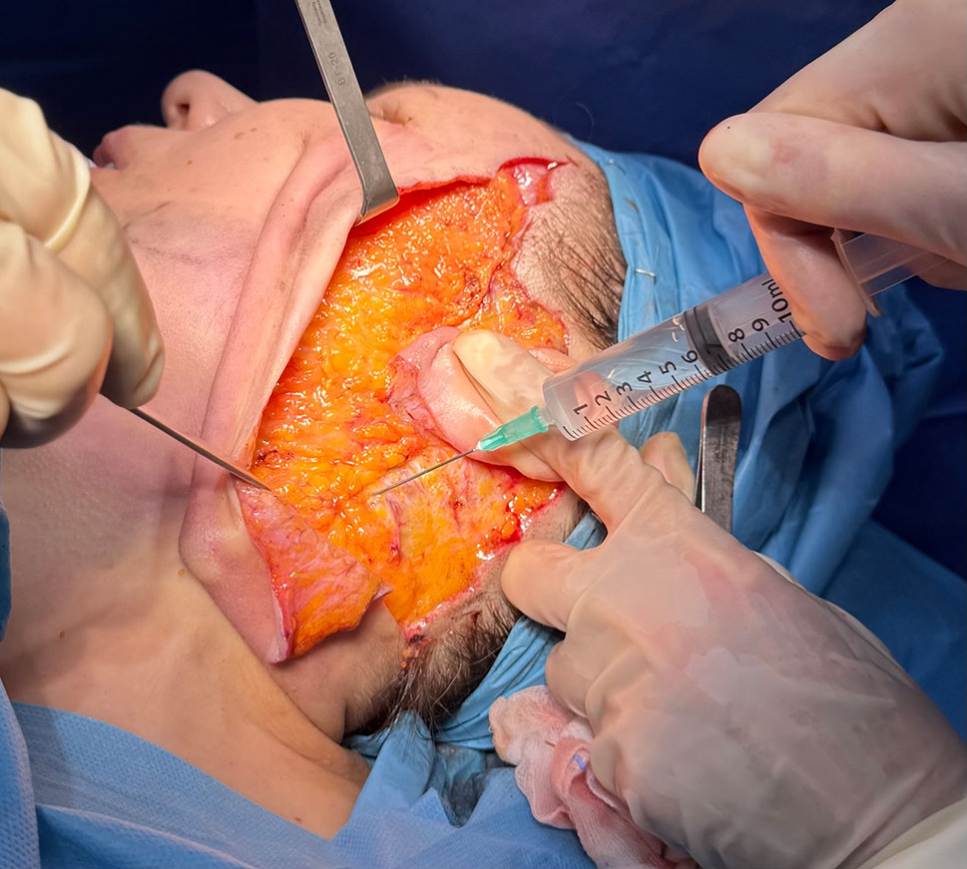
Infiltration of local anesthetic Chirocaine 7.5 mg/mL. Owing to its long half-life, it provides effective postoperative analgesia and contributes to enhanced blood pressure stability

A key preventive measure, performed during the final surgical stage but yielding benefits in the following hours, is the administration of Chirocaine (Levobupivacaine), a long-acting local anesthetic, with efficacy lasting up to 16 h. This ensures adequate analgesia during the immediate postoperative period, reducing the risk of hypertensive spikes triggered by acute pain. Ten ml of Chirocaine 7.5 mg/ml is injected bilaterally at the deep anchoring points of the SMAS flap—specifically, the mastoid periosteum and the periosteum of the posterior third of the zygomatic arch—sites frequently associated with postoperative pain (Fig. 7).

An overview of the discussed concepts is provided in Table 1.

**Table 1:** Multistep protocol to prevent postoperative hematoma.

Preoperative phase	Intraoperative phase	Postoperative phase
Discontinue medications and supplements known to increase bleeding risk—such as aspirin, NSAIDs, and certain herbal products—for at least two weeks prior to surgery	Infiltration of 100–120 cc per side with a solution composed of 220 cc of saline, 20 cc of 3% mepivacaine, 1 mg of epinephrine, and 500 mg of tranexamic acid	Moderately compressive bandage to cover all regions of adipo-cutaneous flap dissection across the face and neck
Transdermal clonidine patch (5 mg) 24 h prior to surgery to achieve perioperative blood pressure stabilization	Fibrin glue (ARTISS, Baxter Healthcare Corp., Deerfield, Illinois; 4 ml)	Strict control of blood pressure, retching, Valsalva maneuvers, and hypertensive spikes
	Meticulous hemostasis while maintaining physiological (normotensive) blood pressure levels	
	Optional placement of a transcutaneous hemostatic net (Auersvald type)	
	Smooth emergence from general anesthesia to prevent retching, Valsalva maneuvers, and peri-extubation hypertensive spikes	
	10 ml vial of Chirocaine 7.5 mg/ml infiltrated where the SMAS flap is anchored deeply	

Overview of the authors’ strategies across pre-, intra-, and postoperative phases aimed at reducing blood accumulation and hematoma risk after rhytidectomy.

Despite adherence to the complete protocol, two cases of postoperative hematoma were recorded over the past five years.

Regarding hematoma management, approximately 70% of all cases recorded over the past 40 years were managed conservatively at the bedside using aspiration, irrigation, local compression, and blood pressure optimization. In the remaining 30%, surgical revision was required to perform open hematoma evacuation and lavage. It is essential to remove all coagulated blood to prevent fibrotic sequelae and the development of retractile, deforming scars.

### Limitations and Strengths

The primary limitation of this study is its retrospective design and the absence of a formal control group. Nonetheless, the exceptionally low hematoma rate (i.e., two patients out of more than 500 cases) achieved over the past five years following the systematic application of the described protocol offers compelling descriptive evidence of efficacy. The large case volume enhances the robustness and clinical relevance of the findings, particularly when compared to prospective studies reporting hematoma rates ranging from 0.2 to 15%. [5, 6]

The study’s main contribution lies in the development of a cohesive, standardized, and reproducible perioperative protocol that integrates multiple evidence-based strategies—including TXA infiltration, fibrin glue, hemostatic net application, and blood pressure management—into a step-by-step guide for clinical use. Additionally, it incorporates detailed perioperative measures often underreported in the literature, such as preoperative clonidine administration and intraoperative use of Chirocaine. While none of these interventions are individually novel, their systematic integration and consistent application represent a valuable model for standardizing best practices in aesthetic surgery.

A further distinctive element is the refined surgical technique employed: a modified High SMAS/Extended SMAS approach with precise vector adjustments and stable anchoring points, which may contribute to improved long-term outcomes.

This study does not aim to be a systematic review or formal comparative study, but rather a practical, experience-based resource, that translates consolidated evidence into a structured and implementable workflow. Its strength lies in its direct applicability to surgical practice, particularly in high-volume settings where consistency, reproducibility, and complication reduction are essential.

## Conclusions

Facelifts have undergone significant evolution over time, aiming to improve aesthetic outcomes and reduce postoperative complications. Among these, hematoma remains one of the most concerning adverse events.

The two primary surgeons have performed over 3000 facelift procedures. Through the consistent application of the techniques and protocols described herein, they have achieved a postoperative hematoma rate of approximately 0.3% to date. The extensive surgical experience supports the effectiveness of these methods, while the high number of procedures performed underscores the reproducibility of the approach in preventing postoperative hematomas.

The adoption of targeted pre-, intra-, and postoperative strategies has proven effective in drastically reducing the incidence of this complication. Optimized blood pressure management, the use of antifibrinolytic agents such as tranexamic acid, the application of fibrin glue to eliminate dead space and enhance tissue adhesion, and the use of the Auersvald hemostatic net, have all contributed to minimizing postoperative hemorrhagic risk.

Available data demonstrate that a multimodal approach is essential to effectively reduce the risk of postoperative hematoma. No single intervention is definitively protective; rather, it is the combination of evidence-based strategies that enhances both safety and efficacy in facelift surgery. The integration of these protocols into routine surgical practice can significantly improve clinical outcomes, reduce morbidity, and facilitate faster, safer patient recovery.
